# A simple DEB-based ecosystem model

**DOI:** 10.1093/conphys/coac057

**Published:** 2022-08-06

**Authors:** Jaap van der Meer, Vincent Hin, Pepijn van Oort, Karen E van de Wolfshaar

**Affiliations:** Wageningen Marine Research, Korringaweg 7, 4401 NT Yerseke, The Netherlands, jaap.vandermeer@wur.nl, +31 317 488105; Aquaculture and Fisheries Group, Wageningen University and Research, Wageningen, The Netherlands; Animal Ecology Group, VU University, Amsterdam, The Netherlands; Wageningen Marine Research, Korringaweg 7, 4401 NT Yerseke, The Netherlands, jaap.vandermeer@wur.nl, +31 317 488105; Wageningen Plant Research, Wageningen, The Netherlands; Wageningen Marine Research, Korringaweg 7, 4401 NT Yerseke, The Netherlands, jaap.vandermeer@wur.nl, +31 317 488105

**Keywords:** canonical community model, Dynamic Energy Budget (DEB) theory, mass transformations, stoichiometry, synthesizing unit, V1-morph

## Abstract

A minimum stoichiometric carbon and nitrogen model of an entire ecosystem based on Dynamic Energy Budget (DEB) theory is presented. The ecosystem contains nutrients, producers, consumers, decomposers and detritus. All three living groups consist of somatic structure and either one (consumers and decomposers) or two (producers) reserve compartments, hence the living matter is described by seven state variables. Four types of detritus are distinguished. As the system is closed for matter, the dynamics of the nutrients carbon dioxide and ammonium follow automatically from the dynamics of the other 11 state variables. All DEB organisms in the model are V1-morphs, which means that surface area of each organism is proportional to volume. The resulting ontogenetic symmetry implies that complicated modelling of size structure is not required. The DEB V1-morph model is explained in detail, and the same holds for the idea of synthesizing units, which plays a key role in DEB modelling. First results of system dynamics are presented.

## Teaser

The main building blocks of each ecosystem model are descriptions of how the physiology and energetics of individual organisms relate to their environment. The well-established Dynamic Energy Budget (DEB) theory provides such descriptions of the uptake, allocation and use of energy and matter by individual organisms in relation to food availability. In this paper we present an ecosystem model, containing a producer, a consumer, a decomposer and various nutrients and forms of detritus, that is entirely based on DEB theory. We estimate model parameters and explore model dynamics.

## 1 Introduction

Human activities often impose direct effects on organisms of high conservation value. The construction of wind farms, for example, puts iconic bird species, such as vultures, eagles and larger seabirds, at risks of collisions with wind turbines. Mortality rates increase, with possible negative effects on population size (Schippers *et al.*, 2020). More subtle indirect effects through changes in the entire food web should, however, not be underestimated. Offshore wind farms in the southern North Sea decrease the sea surface wind speed and affect the stratification development (Christiansen *et al.*, 2022). These changes in the physical environment may alter primary production level with cascading effects through the entire food web, ultimately up to seabird abundance. The use of mathematical ecosystem models is required for the assessment of such indirect effects. This, of course, holds for many other types of human impact, such as eutrophication, pollution or climate change-related seawater warming and acidification.

Models of the metabolism of individual organisms, which describe among other things rates of ingestion, assimilation, maintenance, growth and reproduction in response to the organism’s state and the environment, are the essential building blocks of such ecosystem models. The Dynamic Energy Budget (DEB) theory developed by Kooijman and co-workers (Kooijman, 1986a,b, 2010, Kooijman *et al.*, 2008, Sousa *et al.*, 2008) provides models of organism’s metabolism, which are now widely used at the level of the individual. The standard DEB model, appropriate for species with isomorphic growth, has been tested for over a thousand different species (Marques *et al.*, 2018, van der Meer *et al.*, 2014). The V1-morph DEB model, which is suitable for organisms growing in such way that surface area is more or less proportional to mass, has been used for a variety of micro-organisms (Brandt *et al.*, 2004, Grossowicz *et al.*, 2017, Lika & Papadakis, 2009, Livanou *et al.*, 2019). Despite the availability of such a well-established theoretical framework, most community and ecosystem models still use descriptions of species metabolism that are not derived from a general theory, but are merely based on old habits (Anderson & Mitra, 2010, Babel *et al.*, 2019, Maps & Record, 2020). Such practice hampers generality and transferability and thus slows down scientific progress. In this paper we describe in detail the simplest possible ecosystem (or community, if you like) model that is based on DEB theory. The model should be considered as a basic model that can be extended for specific conservation goals.

This minimum ecosystem model includes a nutrient, a producer, a consumer and a decomposer, and has a closed mass-balance, essential prerequisites of ecosystem models. It is assumed that all species are V1-morphs, which means that area is proportional to volume for an individual organism. DEB theory assumes that some process rates, such as food ingestion rate, are area related, whereas other rates, like maintenance rate, are volume related. But for a V1-morph the area–volume ratio does not change during growth and all rates are therefore implicitly volume-related. Organism size plays no role, a condition sometimes referred to as ontogenetic symmetry (Roos *et al.*, 2013). Take, for example, an organism in the form of a rope that only grows in length. It then does not matter whether the rope is cut in smaller pieces or not, as long the area of the tips is negligibly small. Hence, there is no real difference between individuals and populations and complicated modelling of the size structure of populations is not needed.

We embroider on the work of Kooijman & Nisbet (2000) and Kooijman (2010), who presented a first version of this model, which they called the canonical community model, although they did this in a rather concise way. We extended the model by including the possibility that the producer can also shrink when nutrient or light levels get too low. The main value of our contribution is that we provide an extensive treatment of the underlying ideas and how they translate in a set of differential equations. We also pay attention to parameter estimation and implementation.

Before we discuss the ecosystem model in detail, we first introduce the specifics of the V1-morph model and its link with the standard DEB model for isomorphs, discuss the use of different dimension frameworks (e.g. energy–length or mass–mass) in DEB modelling and explain the idea of synthesizing units (SUs), which play a key role in DEB theory. We assume some basic knowledge of the so-called standard DEB model (Kooijman, 2010), for which gentle introductions are available (van der Meer, 2006b, 2016, 2019).

## 2 V1-morphs

**Fig. 1:**
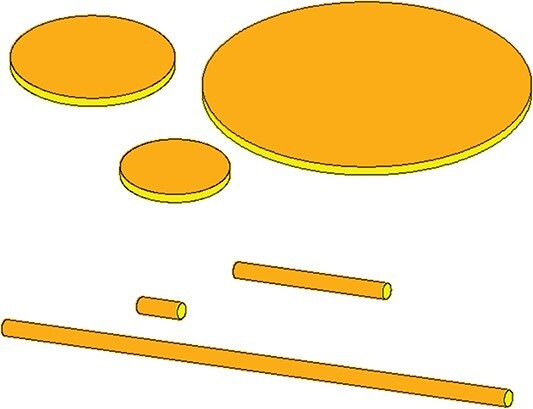
A rope growing in length and a pancake growing in diameter are both V1-morphs. The area responsible for the uptake of resources is coloured orange.

One of the main assumptions in DEB theory is that ingestion and assimilation rates are proportional to the surface area of the structural body of the organism. The standard DEB model deals with isomorphic animals. An isomorphic animal does not change in shape during growth. If two animals have the same shape, then every kind of length measure taken at one individual relates to the same measure taken at the other individual by a constant factor. Isomorphism thus implies that surface area scales with squared length and volume with cubic length. Although most animal species are approximately isomorphic, many other organisms are not. Filamentous fungi form branches, where the uptake of resources occurs at the tip area of each branch and the number of branches is proportional to the volume of the total mycelium (Gow & Gadd, 1995, Trinci, 1974). Such an organism is called a V1-morph in DEB theory, which implies that the scaling factor that links area to volume equals one. Area is thus proportional to volume. Other (theoretical) examples of V1-morphs are organisms in the form of a rope that only grow in length and where the tips do not contribute to the uptake, or organisms in the form of sheets or crusts that only grow in diameter and where the uptake is only at the top or bottom (Fig. 1). Colonies of archaea, cyanobacteria or green algae may also fit this pattern. One specific example is the genus *Pediastrum*.

Organisms that do not adhere to isomorphs can be modelled using a shape correction factor. The shape correction factor is defined as

\begin{align*} \mathcal{M}\left(V\right)=\left(\frac{V}{V_d}\right)^{x-2/3}, \end{align*}

where $V$ is structural volume, $V_d$ is a reference volume required to keep the correct physical dimensions and where the scaling factor $x$ indicates the morph type, having a value of 1 for a V1-morph, 2/3 for an isomorph and 0 for a V0-morph, the latter thus having a constant surface area. The assimilation rate of a V1-morph equals

\begin{align*} \dot{p}_A=\mathcal{M}\left(V\right)\{\dot{p}_{Am}\}V^{2/3}=\left(\frac{V}{V_d}\right)^{1/3}\{\dot{p}_{Am}\}V^{2/3}=\frac{\{\dot{p}_{Am}\}}{V_d^{1/3}}V, \end{align*}

which can be simplified to

\begin{align*} \dot{p}_A=[\dot{p}_{Am}]V. \end{align*}

The parameter $\{\dot {p}_{Am}\}$ is one of the primary parameters of the standard DEB model and stands for the maximum area-specific assimilation rate; the parameter $[\dot {p}_{Am}]$ is the maximum volume-specific assimilation rate.

### 2.1 V1-morph reserve dynamics and structural growth

One consequence of the assumptions of homeostasis that are made in DEB theory ( for a detailed explanation, see Kooijman, 2010) is that reserve density $[E]$, which is the amount of reserves per unit of structural volume, follows first order dynamics. In the case of V1-morphs, the rate at which the reserve density drops down when no assimilation takes place only depends upon the reserve density

(1) \begin{align*}& \frac{\textrm{d}[E]}{\textrm{d}t}=\frac{\dot{p}_A}{V}-\dot{k}_E[E]=[\dot{p}_{Am}]f-\dot{k}_E[E], \end{align*}

where $\dot {p}_A$ is the assimilation rate and $f$ is the scaled functional response indicating the food availability. The proportionality coefficient $\dot {k}_E$ has been given the name ‘specific energy conductance’ and has the physical dimension ‘per time’. The maximum reserve density equals the ratio of the maximum volume-specific assimilation rate to the specific energy conductance $[E_m]=[\dot {p}_{Am}]/\dot {k}_E$.

The reserves $E$ equal the reserve density $[E]$ times the volume $V$. Using equation 1 and the chain rule gives the mobilization rate, which is the rate at which the reserves are mobilized:

(2) \begin{align*}& \dot{p}_C=\dot{k}_E[E]V-[E]\frac{\textrm{d}V}{\textrm{d}t}. \end{align*}

The allocation of the mobilized matter can be simplified if organisms do not produce gonads, but simply divide into two daughter cells. Such dividing organisms are classified as juveniles in DEB terminology. For juveniles, maturity and maturity maintenance are not directly relevant for describing growth. This is a consequence of the $\kappa $-rule of DEB theory, which says that a fixed fraction $\kappa $ of the mobilization rate is spent on growth and somatic maintenance. The remaining fraction is spent on maturity and maturity maintenance. To keep things as simple as possible, we therefore take $\kappa =1$. Surface area-related maintenance costs are irrelevant for V1-morphs, and hence the allocation is given by

(3) \begin{align*}& \dot{p}_C = [E_G]\frac{\textrm{d}V}{\textrm{d}t}+[\dot{p}_M]V, \end{align*}

where $[E_G]$ are the energetic growth costs per unit of growth of structural volume and $[\dot {p}_M]$ are the volume-specific maintenance costs. Substituting equation 2 in equation 3 gives the growth equation

(4) \begin{align*}& \frac{\textrm{d}V}{\textrm{d}t}=\frac{\dot{k}_E [E] - [\dot{p}_M] }{[E]+[E_G]}V. \end{align*}

Under constant food conditions, when the reserve density is in equilibrium and proportional to the scaled functional response, the growth equation simplifies to

(5) \begin{align*}& \frac{\textrm{d}V}{\textrm{d}t}=\frac{[\dot{p}_{Am}]f - [\dot{p}_M]}{ [\dot{p}_{Am}]f \dot{k}_E^{-1}+[E_G]}V=\dot{r}V, \end{align*}

where $\dot {r}$ is the specific growth rate. Hence, the growth rate of the structural volume is proportional to the structural volume itself. V1-morphs show exponential growth at constant food density.

The rate at which the reserves are mobilized is thus

(6) \begin{align*}& \dot{p}_C=\left(\dot{k}_E-\dot{r}\right)[E]V. \end{align*}

### 2.2 A mass–mass framework

So far the V1-morph DEB model has been written in a so-called energy-length framework, but, following Kooijman & Nisbet (2000), the model can easily be re-written in a mass–mass framework (Table 1).

**Table 1:** Primary parameters of the DEB model for V1-morphs, specific for a mass–mass framework. The last column indicates the relationship with the parameters from the energy–length framework that has been replaced.

Symbol	Dimension	Interpretation	Relationship
$j_{EAm}$	$\#\#^{-1}t^{-1}$	Mass-specific maximum assimilation rate	$[\dot {p}_{Am}]=\mu _E[M_V]j_{EAm}$
$y_{EX}$	$\#\#^{-1}$	Yield of reserve on food	$\mu _{AX}=\mu _Ey_{EX}$
$j_{EM}$	$\#\#^{-1}t^{-1}$	Mass-specific maintenance rate	$[\dot {p}_M]=\mu _E[M_V]j_{EM}$
$y_{EV}$	$\#\#^{-1}$	Mass-specific costs of growth	$[E_G]=\mu _E[M_V]y_{EV}$

**Fig. 2:**
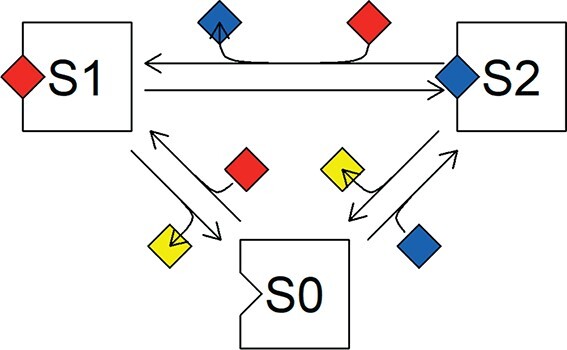
The preference SU. The unit will dissociate a less-preferred object (blue diamond) at the arrival of a preferred object (red diamond), but no product (yellow diamond) is formed at this transition from S2 to S1.

The structural body can be expressed in terms of its mass, or better in terms of the amount of C-atoms, by $M_V=[M_V]V$, where $[M_V]$ is the specific density of the structural body expressed in C-moles per volume. In accordance with the strong homeostasis assumption $[M_V]$ is a constant. Reserve density can be expressed as the number of C-atoms in the reserve per C-atom in the structural body. Reserve mass is related to reserve energy by $M_E=\mu _E^{-1} E$, where $\mu _E$ is the potential energy of the reserves expressed in energy per C-mole. Reserve mass relative to structural mass thus equals $m_E=M_E/M_V=[E] \mu _E^{-1} [M_V]^{-1}$. Within a mass–mass framework the DEB equation for reserve dynamics of V1-morphs thus looks like

\begin{align*} \frac{\textrm{d}m_E}{\textrm{d}t}=\frac{\textrm{d}[E]}{\textrm{d}t}\frac{1}{\mu_E[M_V]}=\frac{[\dot{p}_{Am}]}{\mu_E[M_V]}f -\dot{k}_E\frac{[E]}{\mu_E[M_V]}, \end{align*}

which can be re-written as

(7) \begin{align*}& \frac{\textrm{d}m_E}{\textrm{d}t}=j_{EAm}f-\dot{k}_Em_E, \end{align*}

where $j_{EAm}$ is the maximum mass-specific assimilation rate expressed in C-mole reserve per C-mole structure per time. Growth is given by

(8) \begin{align*}& \frac{\textrm{d}M_V}{\textrm{d}t}=\frac{\textrm{d}V}{\textrm{d}t}[M_V]= \frac{\dot{k}_E m_E - j_{EM}}{m_E+y_{EV}}M_V, \end{align*}

where $j_{EM}=[\dot {p}_M]\mu _E^{-1}[M_V]^{-1}$ refers to the mass-specific maintenance costs, expressed in C-mole reserve per C-mole structure per time, and $y_{EV}=[E_G]\mu _E^{-1}[M_V]^{-1}$ gives the mass-specific costs of growth, expressed in C-mole reserve per C-mole structure. The growth equation has the form $\textrm {d}M_V/\textrm {d}t=\dot {r}M_V$, where $\dot {r}$ is the specific growth rate. Reserves are mobilized at a rate $ (\dot {k}_E-\dot {r} )m_EM_V$.

**Fig. 3:**
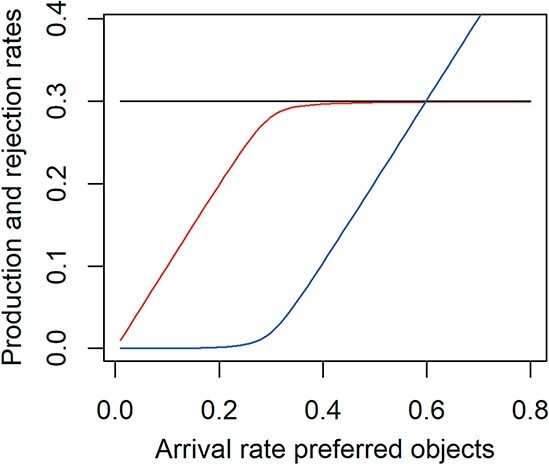
A demand-driven preference SU has a constant product formation rate that equals the demand rate (black horizontal line), but the rate of product formation on the basis of the preferred substrate (red line) depends on the arrival rate of the preferred substrate $\dot {J}_1$, horizontal axis. The difference between the black and red lines gives the rate of product formation on the basis of the less-preferred substrate, whose arrival rate is constant and equal as or larger than the demand rate. The blue line gives the rejected flux of preferred substrate. At high arrival rate of the preferred substrate hardly any less-preferred substrate is used.

## 3 Synthesizing units

DEB theory makes use of so-called synthesizing units or SUs. The idea of SUs could be formalized as a continuous-time Markov process, which is a stochastic process having the property that the future state of the unit (for example empty or busy) only depends upon the present and is independent of the past (Ross, 1989). Objects arrive at an SU by means of a Poisson process, which means that the number of arrivals in any interval is proportional to the length of the interval, which is equivalent to the assumption that the interarrival time is exponentially distributed (Ross, 1989). When the unit is empty, an arriving object is accepted and used by the unit for the synthesis of some product. But arriving objects are rejected in case the unit is busy with handling a previously arrived object. The time after arrival that it takes the unit to produce the product can be described as a stochastic process too, but might also be defined as a fixed time period. Different types of objects can be involved and these types are either substitutable, which means that none of them is essential for product formation, or non-substitutable, which means that all types are needed in the production process. The ecosystem model presented here uses several types of SUs. The first SU type handles one or more substitutable objects, which are some type of substrate. The second type receives two substrates, which are also substitutable, but in this case the unit has a preference for one substrate above the other. A specific type of this preference unit concerns the case when the rate of product formation is demand-driven, which means that it is constrained at some constant value. The third SU type receives two or more non-substitutable substrates, which implies that all types are required for product formation.

### 3.1 SU with one or more substitutable substrates

First consider an SU that only uses one type of substrate. The unit is either empty (a fraction $S_0/S$ of all units is empty), or contains a substrate (fraction $S_1/S$), where $S$ is the overall density of units. If a unit is empty it will accept an arriving substrate. The following two differential equations describe the dynamics of the number of units in each of the two states:

\begin{eqnarray*} \frac{dS_0}{dt}&=&-\dot{b}XS_0+\dot{k}S_1\\ \frac{dS_1}{dt}&=&\dot{b}XS_0-\dot{k}S_1,\end{eqnarray*}

where $\dot {J}_i=\dot {b}X$ is the arrival rate of substrates and $\dot {k}$ is the dissociation parameter. These differential equations are equal to zero at equilibrium, sometimes called pseudo-equilibrium because $X$ is assumed constant. This reveals $S_1^*=\frac {\dot {b}}{\dot {k}}XS_0^*$, where $S_0^*$, and $S_1^*$ refer to the equilibrium densities. Since $S_0^*+S_1^*=S$ it follows that $S_1^*=\frac {\dot {b}}{\dot {k}}X\left (S-S_1^*\right )$, and thus $\left (1+\frac {\dot {b}}{\dot {k}}X\right )S_1^*=\frac {\dot {b}}{\dot {k}}XS$. The overall processing rate per SU equals the dissociation parameter times the fraction of units of type 1, and is thus given by

\begin{align*} \dot{J}=\dot{k}\frac{S_1^*}{S}=\frac{\dot{b}X}{1+\frac{\dot{b}}{\dot{k}}X}. \end{align*}

Ecologists will recognize that the result is equivalent to Holling’s disc equation, also called the type II functional response equation, where $\dot {b}$ is the area of discovery, and $1/\dot {k}$ the handling time. The equation is often written as

\begin{align*} \dot{J}=\dot{k}\frac{S_1^*}{S}=\dot{k}\frac{X/K}{1+X/K}=\dot{k}\frac{X}{K+X}, \end{align*}

where $\dot {k}$ is the maximum processing rate and where the half-saturation coefficient $K$ is equivalent to $\frac {\dot {k}}{\dot {b}}$.

Similarly, an SU that uses multiple types of substitutable substrates results in the equivalence of Holling’s type II functional response equation for multiple prey. For two substrates, the unit is either empty, contains a substrate of type 1 or of type 2. The three differential equations that describe the dynamics of the density of units in each of the three states are

\begin{eqnarray*} \frac{dS_0}{dt}&=&-\left(\dot{b}_1X_1+\dot{b}_2X_2\right)S_0+\dot{k}_1S_1+\dot{k}_2S_2\\ \frac{dS_1}{dt}&=&\dot{b}_1X_1S_0-\dot{k}_1S_1\\ \frac{dS_2}{dt}&=&\dot{b}_2X_2S_0-\dot{k}_2S_2, \end{eqnarray*}

where $\dot {J}_i=\dot {b}_iX_i$ is the arrival rate and $\dot {k}$ the dissociation parameter for substrate $i$. At equilibrium

\begin{eqnarray*} S_0^*&=&S_0^*\\ S_1^*&=&\frac{\dot{b}_1}{\dot{k}_1}X_1S_0^*\\ S_2^*&=&\frac{\dot{b}_2}{\dot{k}_2}X_2S_0^*\\ S_0^*+S_1^*+S_2^*&=&\left(1+\frac{\dot{b}_1}{\dot{k}_1}X_1+\frac{\dot{b}_2}{\dot{k}_2}X_2\right)S_0^*\\ \end{eqnarray*}

and since $S_0^*+S_1^*+S_2^*=S$, it follows that the overall processing rate for substrate $i$ is given by

\begin{align*} \dot{J_i}=\dot{k}_i\frac{S_i^*}{S}=\frac{\dot{b}_iX_i}{1+\frac{\dot{b}_1}{\dot{k}_1}X_1+\frac{\dot{b}_2}{\dot{k}_2}X_2}=\dot{k}_i\frac{X_i/K_i}{1+X_1/K_1+X_2/K_2}. \end{align*}

### 3.2 Preference SU with two substitutable substrates

Consider an SU that can use two types of substrates, but has a preference for one type. The unit is either empty (a fraction $S_0/S$ of all units is empty), contains the preferred substrate (fraction $S_1/S$) or the less appreciated substrate (fraction $S_2/S$). If the unit is empty it will always accept both types once they arrive. If the unit contains the less appreciated substrate 2 and a preferred substrate 1 arrives, it will delete type 2 and replace it by the preferred type 1 (Fig.2). The following differential equations describe the system:

\begin{eqnarray*} \frac{dS_0}{dt}&=&-\left(\dot{b}_1X_1+\dot{b}_2X_2\right)S_0+\dot{k}_1S_1+\dot{k}_2S_2\\ \frac{dS_1}{dt}&=&\dot{b}_1X_1\left(S_0+S_2\right)-\dot{k}_1S_1\\ \frac{dS_2}{dt}&=&\dot{b}_2X_2S_0-\left(\dot{b}_1X_1+\dot{k}_2\right)S_2, \end{eqnarray*}

where $\dot {J}_i=\dot {b}_iX_i$ is the arrival rate and $\dot {k}_i$ is the dissociation parameter for substrate $i$. The dynamics of the preferred substrate 1 follow exactly Holling’s disc equation, as it does not matter whether the unit is in state 0 or state 2, in both cases it will accept an arriving item of type 1. In equilibrium

\begin{align*} \frac{S_1^*}{S}=\frac{\dot{J}_1}{\dot{k}_1+\dot{J}_1}. \end{align*}

Noting that $S_0=S-S_1-S_2$ and setting the third differential equation equal to zero, gives

\begin{align*} \dot{b}_2X_2\left(S-S_1^*-S_2^*\right)-\left(\dot{b}_1X_1+\dot{k}_2\right)S_2^*=0 \end{align*}

from which the equilibrium density for state 2 follows

\begin{align*} \frac{S_2^*}{S}=\frac{\dot{J}_2(1-S_1^*/S)}{\dot{k}_2+\dot{J}_1+\dot{J}_2}= \frac{\dot{k}_1\dot{J}_2}{(\dot{k}_1+\dot{J}_1)(\dot{k}_2+\dot{J}_1+\dot{J}_2)}, \end{align*}

where $\dot {J}_2=\dot {b}_2X_2$.

For each unit, the product $P$ is delivered with a rate

\begin{align*} \dot{J}_P=y_1\dot{k}_1\frac{S_1}{S}+y_2\dot{k}_2\frac{S_2}{S}, \end{align*}

where $y_i$ gives the number of product particles formed per substrate particle $i=1,2$.

In case of ‘demand kinetics’ the product delivery rate $\dot {J}_P$ has to be constant $\dot {J}_P=\dot {k}_P$ and the dissociation parameters $\dot {k}_1$ and $\dot {k}_2$ will be tuned accordingly. The only requirement is that the ratio between the two parameters remains constant, given by the so-called preference parameter $\rho =\dot {k}_2/\dot {k}_1$ (Fig.3).

In equilibrium, using $\dot {k}_1=\dot {k}$ and $\dot {k}_2=\rho \dot {k}$, the delivery rate is given by

\begin{align*} \frac{y_1\dot{k}\dot{J}_1}{\dot{k}+\dot{J}_1}+\frac{y_2\rho\dot{k}^2\dot{J}_2}{(\dot{k}+\dot{J}_1)(\rho \dot{k}+\dot{J}_1+\dot{J}_2)}=\dot{k}_P \end{align*}

and the dissociation parameter $\dot {k}$ can be solved from this equation, which can be re-written as a quadratic equation of the form $A\dot {k}^2+B\dot {k}+C=0$, where

\begin{eqnarray*} A&=&\rho\left(y_1\dot{J}_1+y_2\dot{J}_2-\dot{k}_P\right)\\ B&=& y_1\dot{J}_1(\dot{J}_1+\dot{J}_2)-(\dot{J}_1\rho+\dot{J}_1+\dot{J}_2)\dot{k}_P\\ C&=&-\dot{J}_1(\dot{J}_1+\dot{J}_2)\dot{k}_P \end{eqnarray*}

and where the solution, which always has to be positive, equals

\begin{align*} \dot{k}=\frac{-B+\sqrt{B^2-4AC}}{2A}. \end{align*}

An equivalent approach is to assume that $\dot {k}_1=\dot {k}_P/\theta $ and to solve the equation

\begin{align*} \frac{y_1\frac{\dot{k}_P}{\theta}\dot{J}_1}{\frac{\dot{k}_P}{\theta}+\dot{J}_1}+\frac{y_2\rho(\frac{\dot{k}_P}{\theta})^2\dot{J}_2}{(\frac{\dot{k}_P}{\theta}+\dot{J}_1)(\rho\frac{\dot{k}_P}{\theta}+\dot{J}_1+\dot{J}_2)}=\dot{k}_P \end{align*}

for $\theta $. This gives

\begin{align*} \frac{y_1\dot{k}_P\dot{J}_1}{\dot{k}_P+\dot{J}_1\theta}+\frac{y_2\rho\dot{k}_P^2\dot{J}_2}{(\dot{k}_P+\dot{J}_1\theta)(\rho \dot{k}_P+\dot{J}_1\theta+\dot{J}_2\theta)}=\dot{k}_P \end{align*}

and

\begin{eqnarray*} A&=&\dot{J}_1(\dot{J}_1+\dot{J}_2)\\ B&=&-y_1\dot{J}_1(\dot{J}_1+\dot{J}_2)+(\dot{J}_1+\dot{J}_2)\dot{k}_P+\dot{J}_1\rho\dot{k}_P\\ C&=&\rho\dot{k}_P^2-(y_1\dot{J}_1+y_2\dot{J}_2)\rho\dot{k}_P \end{eqnarray*}

Kooijman (2010) uses still another approach, with $x=S_1^*/(y_2\rho S_2^*)$ as the unknown of a quadratic equation. All three approaches reveal the same result. Below, the first approach will be followed.

### 3.3 SU with two or more non-substitutable substrates

**Fig. 4:**
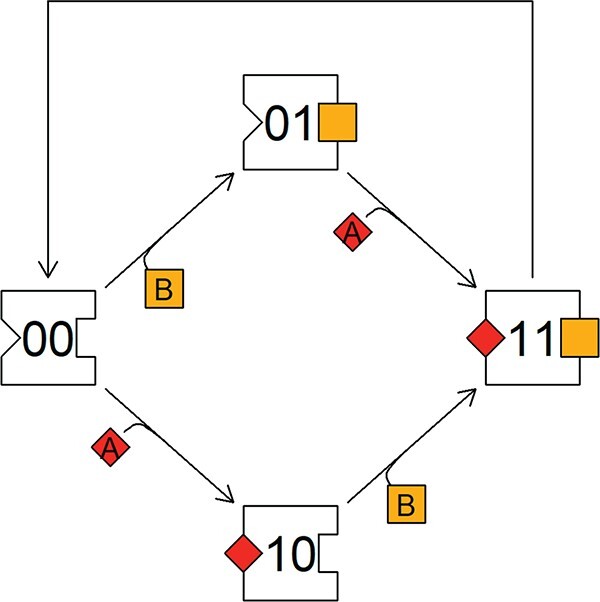
In parallel processing of two non-substitutable substrates, the binding of one type of substrate does not interfere with that of the other. An SU that requires two substrates binds these in a random order, depending upon the order of arrival. When both are bound, the busy unit $11$ will produce a product and then return to the empty state $00$.

Consider, for example, an SU that requires two substrates, one of type A and one of type B, to produce a product P. Suppose further that the binding of one type does not interfere with that of the other, which may be called parallel processing. The density of empty units is given by $S_{00}$, the density of those with only A bound by $S_{10}$, with only B by $S_{01}$ and if both A en B are bound by $S_{11}$ (Fig. 4). The following differential equations apply:

\begin{eqnarray*} \frac{\textrm{d}S_{00}}{\textrm{d}t}&=&-\left(\dot{b}_AX_A+\dot{b}_BX_B\right)S_{00}+\dot{k}S_{11}\\ \frac{\textrm{d}S_{10}}{\textrm{d}t}&=&\dot{b}_AX_AS_{00}-\dot{b}_BX_BS_{10}\\ \frac{\textrm{d}S_{01}}{\textrm{d}t}&=&\dot{b}_BX_BS_{00}-\dot{b}_AX_AS_{01}\\ \frac{\textrm{d}S_{11}}{\textrm{d}t}&=&\dot{b}_BX_BS_{10}+\dot{b}_AX_AS_{01}-\dot{k}S_{11}. \end{eqnarray*}

In equilibrium,

\begin{eqnarray*} S_{00}^*&=&\frac{1}{x_A+x_B}S_{11}^*\\ S_{01}^*&=&\frac{x_A}{x_B}S_{00}^*=\frac{x_A}{x_B\left(x_A+x_B\right)}S_{11}^*\\ S_{10}^*&=&\frac{x_B}{x_A}S_{00}^*=\frac{x_B}{x_A\left(x_A+x_B\right)}S_{11}^*,\\ \end{eqnarray*}

where $x_A$ and $x_B$ are scaled substrate densities, $x_A=\frac {\dot {b}_A}{\dot {k}}X_A=X_A/K_A$ and $x_B=\frac {\dot {b}_B}{\dot {k}}X_B=X_B/K_B$. As the equilibrium densities for all states of the unit are expressed in terms of the density of the busy unit, it is easy to arrive at the process rate per unit (equivalent to the production rate of product C), which, as before, equals $\dot {k}$ times the fraction of busy units

\begin{eqnarray*} \dot{J}_C&=&\dot{k}\frac{S_{11}^*}{S_{00}^*+S_{01}^*+S_{10}^*+S_{11}^*}\\ &=&\dot{k}\frac{1}{\begin{array}{c}\left(x_A+x_B\right)^{-1}+x_Ax_B^{-1}\left(x_A+x_B\right)^{-1}\\
+x_Bx_A^{-1}\left(x_A+x_B\right)^{-1}+1\end{array}}. \end{eqnarray*}

This expression can (after some algebraic manipulation) be simplified to

\begin{align*} \dot{J}_C=\dot{k}\frac{1}{1+x_A^{-1}+x_B^{-1}-\left(x_A+x_B\right)^{-1}} \end{align*}

or

\begin{align*} \dot{J}_C=\frac{1}{\dot{J}_{Cm}^{-1}+\dot{J}_A^{-1}+\dot{J}_B^{-1}-\left(\dot{J}_A+\dot{J}_B\right)^{-1}}, \end{align*}

where $\dot {J}_{Cm}=\dot {k}$ is the maximum process rate and $\dot {J}_A=\dot {b}_AX_A$ and $\dot {J}_B=\dot {b}_BX_A$ are the rates at which substrates A and B, respectively, arrive at each server. Thus, $\dot {J}_{Cm}^{-1}$ is the expected ‘servicing’ time and $\dot {J}_A^{-1}$ and $\dot {J}_B^{-1}$ are the expected interarrival times of type A and B substrates, respectively.

It can be shown that if both types of substrates can not be bound simultaneously (i.e. the binding of one can only start when the other is already bound) and when the order in which they arrive is important, say first A and then B, (this is called sequential processing) that the process rate decreases to

\begin{align*} \dot{J}_C=\frac{1}{\dot{J}_{Cm}^{-1}+\dot{J}_A^{-1}+\dot{J}_B^{-1}}. \end{align*}

The expected processing time $\dot {J}_C^{-1}$ is then simply the sum of the expected ‘servicing’ time $\dot {J}_{Cm}^{-1}$ and the expected interarrival times of the substrates (here $\dot {J}_A^{-1}$ plus $\dot {J}_B^{-1}$).

**Fig. 5:**
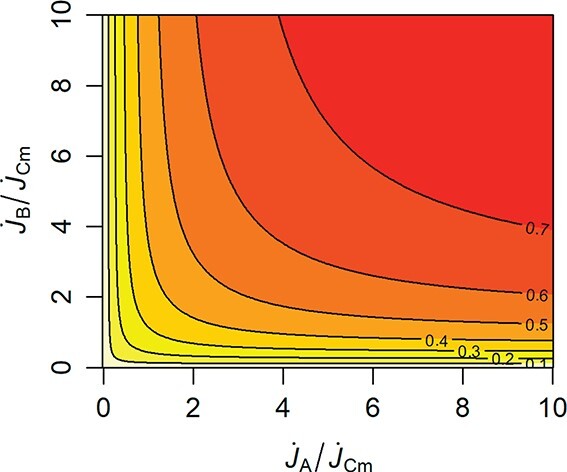
Contours of the production rate as a function of the rates at which substrate A and B arrive, for a parallel processing SU that requires two non-substitutable substrates to produce a product C (as in Fig. 4).

The parallel processing SU described above behaves very much like a minimum operator (Fig. 5), where it is assumed, following Liebig’s law, that the processing rate is only limited by one type of substrate, the so-called limiting substrate

\begin{align*} \dot{J}_C=\dot{J}_{Cm}^{-1}\max\left(\frac{X_A}{K_A+X_A},\frac{X_B}{K_B+X_B}\right). \end{align*}

In ecology, the use of the minimum operator in growth models has been popularized by Tilman (1988). The SU approach should, however, be preferred, not only for its elegance and greater realism, but also because it prevents numerical problems related to the stepwise change of the minimum operator, in further applications, for example in population models.

## 4 The canonical ecosystem model

The canonical ecosystem model described here is the most simple DEB-based model for V1-morphs, formulated in a mass–mass framework and keeping track of carbon and nitrogen fluxes. It contains apart from the nutrients carbon dioxide and ammonia, three living (producers, consumers and decomposers) and one non-living group (detritus). Each living group is characterized by a structure and by two (producers) or one (the other two groups) type of reserves. Detritus is split up in four types, depending on the origin, which can either be consumer faeces as a result of eating producers, consumer faeces from eating decomposers, consumer dead structure or consumer dead reserves. The model has therefore 13 state variables (Table 2). As the system is closed for matter, the dynamics of the nutrients follow automatically from the dynamics of the other 11 state variables. Fluxes from and to these 11 state variables are presented in a flux matrix (Table 3). Parameters and conversion coefficients are not explained in the text, but given in Tables 4, 5 and 6.

**Table 2:** State variables of the canonical ecosystem model. The first letter of the index stands for the type of compound: P, faeces or dead material; V, structural mass; E, reserves; C, carbon dioxide; and N, ammonia. The second letter stands for the origin of the faeces: P, producer; D, decomposer; V, consumers’ structure; E, consumers’ reserves or for the group; C, consumer; P, producer; and D, decomposer.

Group	Compound	Variable	Scaled variable
Detritus	Producer faeces	$M_{PP}$	$x_{PP}=M_{PP}/K_{PP}$
Detritus	Decomposer faeces	$M_{PD}$	$x_{PD}=M_{PD}/K_{PD}$
Detritus	Dead consumer structure	$M_{PV}$	$x_{PV}=M_{PV}/K_{PV}$
Detritus	Dead consumer reserve	$M_{PE}$	$x_{PE}=M_{PE}/K_{PE}$
Consumer	Structure	$M_{VC}$	
Consumer	Reserve	$M_{EC}$	$m_{EC}=M_{EC}/M_{VC}$
Producer	Structure	$M_{VP}$	$x_P=M_{VP}/K_{VP}$
Producer	Reserve 1	$M_{E_1P}$	$m_{E_1P}=M_{E_1P}/M_{VP}$
Producer	Reserve 2	$M_{E_2P}$	$m_{E_2P}=M_{E_2P}/M_{VP}$
Decomposer	Structure	$M_{VD}$	$x_D=M_{VD}/K_{VD}$
Decomposer	Reserve	$M_{ED}$	$m_{ED}=M_{ED}/M_{VD}$
Nutrient	Carbon dioxide	$M_C$	
Nutrient	Ammonia	$M_N$	

### 4.1 Consumers

Consumers predate on both producers and decomposers and the overall predation rate is given by the processing rate of an SU with two substitutable substrates, or in the terminology of an animal ecologist by a Holling’s type II functional response with two prey types. The consumption or removal rate of producer structure (in terms of a flux measured in C-moles) is given by

(9) \begin{align*}& \dot{J}_{VP,A_1C}=-M_{VC}j_{VP,AC,m}\frac{x_P}{1+x_P+x_D}, \end{align*}

where the minus sign indicates that matter is subtracted from a compartment, in this case from producer structure. All fluxes in the model that are subtracted from one of the 11 compartments have a negative sign, and those that are directed to one of these compartments have a positive sign. The removal of producers by predating consumers or the consumption of detritus by decomposers, for example, are represented by negative fluxes. The same holds for fluxes that describe the mobilization of reserves or structure. Fluxes directed towards reserves (assimilation) or towards structure (growth) have a positive sign. But beware that a general description of a flux, for example one indicating the required maintenance costs, without being specific where the flux comes from or goes to, is given as a positive figure.

The consumption of producer structure goes hand-in-hand with the consumption of the two producer reserve types

(10) \begin{align*}& \dot{J}_{E_iP,A_1C}=m_{E_iP}\dot{J}_{VP,A_1C} \end{align*}

for $i\in \{1,2\}$. Part of the consumed matter ends up in the reserves of the consumer

(11) \begin{align*}& \dot{J}_{EC,A_1C}=-\left(y_{EC,VP}+\sum_{i=1}^2{y_{EC,E_iP}m_{E_iP}}\right)\dot{J}_{VP,A_1C}. \end{align*}

Note that the minus sign will turn the negative consumption fluxes into a positive assimilation flux. Another part of the consumed matter ends up in the producer faeces fraction of the detritus pool

(12) \begin{align*}& \dot{J}_{PP,A_1C}=-y_{PP,VP}\dot{J}_{VP,A_1C}. \end{align*}

Similarly for the consumption of decomposers, which only have one type of reserves, by consumers

(13) \begin{align*}& \dot{J}_{VD,A_2C}=-M_{VC}j_{VD,AC,m}\frac{x_D}{1+x_P+x_D} \end{align*}

(14) \begin{align*}& \dot{J}_{ED,A_2C}=m_{ED}\dot{J}_{VD,A_2C} \end{align*}

(15) \begin{align*}& \dot{J}_{EC,A_2C}=-\left(y_{EC,VD}+y_{EC,ED}m_{ED}\right)\dot{J}_{VD,A_2C} \end{align*}

(16) \begin{align*}& \dot{J}_{PD,A_2C}=-y_{PD,VD}\dot{J}_{VD,A_2C}. \end{align*}

The consumers mobilize their reserves at a rate (negative sign) equal to

\begin{align*} \dot{J}_{EC,CC}=-(\dot{k}_{EC}-\dot{r}_{VC,GC})m_{EC}M_{VC}, \end{align*}

where the term $\dot {r}_{VC,GC}m_{EC}M_{VC}$ is needed to avoid dilution by growth of the reserve density (compare with equation 6). The instantaneous growth rate equals

\begin{align*} \dot{r}_{VC,GC}=\frac{\dot{k}_{EC}m_{EC}-j_{EC,MC}}{m_{EC}+y_{EC,VC}}, \end{align*}

where $j_{EC,MC}$ is the mass-specific maintenance rate of the consumers (see also equation 8).

**Table 3:** The flux matrix $\dot {J}^T$ showing the relevant equation for each flux. Columns refer to state variables (see also Table 2); rows refer to the various processes, where (first letter) A stands for assimilation, G for growth, D for dissipation, H for death and (second letter) C for consumer, P for producer and D for decomposer. A minus sign indicates a disappearing flux.

	$PP$	$PD$	$PV$	$PE$	$VC$	$EC$	$VP$	$E_1P$	$E_2P$	$VD$	$ED$
$A_1C$	12					11	-9	-10	-10		
$A_2C$		16				15				-13	-14
$GC$					20	-19					
$DC$					-18	-17					
$HC$			21	22	-21	-22					
$A_1P$								23			
$A_2P$									24		
$GP$							25	-26	-26		
$DP$							-28	-27	-27		
$A_1D$	-29										30
$A_2D$		-29									30
$A_3D$			-29								30
$A_4D$				-29							30
$GD$										32	-31
$DD$										-34	-33

**Table 4:** Parameters of the DEB model for the water flea *D. magna*. Original isomorph parameters (upper part), V1-morph parameters for energy–length (middle part) and mass–mass frameworks (lower part).

Parameter	Dimension	Description	*D. magna*
$\{\dot {p}_{Am}\}$	J cm$^{-2}$ d$^{-1}$	Area-spec. max. assimilation	313
$\dot {v}$	cm d$^{-1}$	Energy conductance	0.186
$[\dot {p}_M]$	J cm$^{-3}$ d$^{-1}$	Volume-spec. maintenance	1200
$[E_G]$	J cm$^{-3}$	Volume-spec. cost of growth	4400
$\kappa _X$	-	Digestion efficiency	0.9
$L_d=V_d^{1/3}$	cm	Reference structural length	0.07
$[\dot {p}_{Am}]$	J cm$^{-3}$d$^{-1}$	Volume-spec. max. assimilation	4471
$\dot {k}_E$	d$^{-1}$	Spec. energy conductance	2.66
$j_{EAm}$	C-mole C-mole$^{-1}$ d$^{-1}$	Mass-spec. max. consumption	1.40
$j_{EM}$	C-mole C-mole$^{-1}$ d$^{-1}$	Mass-spec. maintenance	0.337
$y_{EV}$	C-mole C-mole$^{-1}$	Mass-spec. cost of growth	1.237

Usually, the mobilized energy from the reserves is first spent on maintenance, and the rest goes to growth. The maintenance costs, when paid from the reserves, equal $\dot {J}_{EC,MC}=j_{EC,MC}M_{VC}$. However, it might be that the mobilized flux from the reserves is not sufficient to pay the required maintenance. It is therefore assumed that maintenance can also be paid from a flux that comes from structure, and thus allowing shrinkage of structure. The maintenance costs, when paid from structure equal $\dot {J}_{VC,MC}=j_{VC,MC}M_{VC}$. When reserves are transformed into structure, it is assumed that $y_{EC,VC}$ C-moles of reserve are needed to create one C-mole of structure. For convenience, it is also assumed that maintenance, when paid from reserves, requires a fraction $y_{EC,VC}$ more C-moles than when it is paid from structure. Hence, the mobilized flux from structure can be expressed in terms of C-moles of reserves by multiplication with $y_{EC,VC}$.

It is assumed that the two fluxes, one containing reserve substrate and the other structure substrate, are directed towards a demand-driven preference SU that has a preference for reserves above structure. The demand is equivalent to the overall maintenance costs and equals, in terms of reserve C-moles, $\dot {J}_{EC,MC}=j_{EC,MC}M_{VC}$. The mobilized flux from structure can for convenience be taken just as large as to be able to pay for all maintenance. It is, in terms of C-moles of structure, given by

\begin{align*} \dot{J}_{VC,CC}=-j_{VC,CC}M_{VC}. \end{align*}

It is just sufficient for paying all maintenance, which implies that

\begin{align*} j_{VC,CC}=j_{VC,MC}=j_{EC,MC}/y_{EC,VC}. \end{align*}

The work of the preference SU results in a rate of maintenance costs paid from reserves (using the notation of the preference SU as used in Preference SU section and given as a positive flux) equal to

\begin{align*} \dot{J}_{EC}^{MC}=\frac{y_1\dot{k}\dot{J}_1}{\dot{k}+\dot{J}_1}, \end{align*}

where the unknown dissociation parameter is given by

\begin{align*} \dot{k}=\frac{-B+\sqrt{B^2-4AC}}{2A} \end{align*}

with the coefficients $A$, $B$ and $C$ as given earlier in Preference SU section, and with $\dot {J}_1=-\dot {J}_{EC,CC}$, $\dot {J}_2=-y_{EC,VC}\dot {J}_{VC,CC}$, $\dot {k}_p=\dot {J}_{EC,MC}$, $y_1=1$ and $y_2=1$.

**Table 5:** Parameters of the canonical community model.

Name	Unit	Value	Comment	Source
$K_{PP}$	$\mu $ mole C/L	0.1	Half-saturation constant of d~etritus consumption by decomposer	Lorkowski *et al.* (2012)
$K_{PD}$	$\mu $ mole C/L	0.1	Half-saturation constant of d~etritus consumption by decomposer	Lorkowski *et al.* (2012)
$K_{PV}$	$\mu $ mole C/L	0.1	Half-saturation constant of d~etritus consumption by decomposer	Lorkowski *et al.* (2012)
$K_{PE}$	$\mu $ mole C/L	0.1	Half-saturation constant of d~etritus consumption by decomposer	Lorkowski *et al.* (2012)
$K_{VP}$	$\mu $ mole C/L	2	Half-saturation constant of producer consumption by consumer	Kooijman & Nisbet (2000)
$K_{VD}$	$\mu $ mole C/L	5	Half-saturation constant of decomposer consumption by consumer	Kooijman & Nisbet (2000)
$\dot {J}_{K,L_1}$	MJ m$^{-2}$ d$^{-1}$	25	Half-saturation constant of light assimilation by producer reserve 1	
$\dot {J}_{K,L_2}$	MJ m$^{-2}$ d$^{-1}$	22	Half-saturation constant of light assimilation of producer reserve 2	
$K_{C_1}$	$\mu $ mole C/L	3.2	Half-saturation constant of carbon consumption of producer reserve 1	Lorena *et al.* (2010)
$K_{C_2}$	$\mu $ mole C/L	6.4	Half-saturation constant of carbon consumption	Lorena *et al.* (2010)
$K_{N_2}$	$\mu $ mole N/L	0.43	Half-saturation constant of nitrogen consumption	Lorena *et al.* (2010)
$j_{VP,AC,m}$	mole mole$^{-1}$d$^{-1}$	1.40	Maximum consumption rate of producer by consumer	Kooijman & Gergs (2019)
$j_{VD,AC,m}$	mole mole$^{-1}$d$^{-1}$	0.70	Maximum consumption rate of decomposer by consumer	Kooijman & Gergs (2019)
$j_{E_1P,A_1P,m}$	mole mole$^{-1}$d$^{-1}$	5.1	Maximum synthesis rate of producer reserve 1	Lorena *et al.* (2010)
$j_{E_2P,A_2P,m}$	mole mole$^{-1}$d$^{-1}$	1.0	Maximum synthesis rate of producer reserve 2	Lorena *et al.* (2010)
$j_{PP,A_1D,m}$	mole mole$^{-1}$d$^{-1}$	1.4	Maximum uptake rate of detritus pool 1 by decomposer	Lorkowski *et al.* (2012)
$j_{PD,A_2D,m}$	mole mole$^{-1}$d$^{-1}$	1.4	Maximum uptake rate of detritus pool 2 by decomposer	Lorkowski *et al.* (2012)
$j_{PV,A_3D,m}$	mole mole$^{-1}$d$^{-1}$	1.4	Maximum uptake rate of detritus pool 3 by decomposer	Lorkowski *et al.* (2012)
$j_{PE,A_4D,m}$	mole mole$^{-1}$d$^{-1}$	1.4	Maximum uptake rate of detritus pool 4 by decomposer	Lorkowski *et al.* (2012)
$j_{EC,MC}$	mole mole$^{-1}$d$^{-1}$	0.337	Maintenance rate of consumer	Kooijman & Gergs (2019)
$j_{E_1P,MP}$	mole mole$^{-1}$d$^{-1}$	0.054	Maintenance rate of producer from reserve 1	Lorena *et al.* (2010)
$j_{E_2P,MP}$	mole mole$^{-1}$d$^{-1}$	0.012	Maintenance rate of producer from reserve 2	Lorena *et al.* (2010)
$j_{ED,MD}$	mole mole$^{-1}$d$^{-1}$	0.192	Maintenance rate of decomposer	Eichinger *et al.* (2009)
$\dot {k}_{EC}$	d$^{-1}$	2.66	Specific energy conductance consumer	Kooijman & Gergs (2019)
$\dot {k}_{E_1P}$	d$^{-1}$	5.2	Specific energy conductance producer reserve 1	Grossowicz *et al.* (2017)
$\dot {k}_{E_2P}$	d$^{-1}$	5.2	Specific energy conductance producer reserve 2	Grossowicz *et al.* (2017)
$\dot {k}_{ED}$	d$^{-1}$	14.5	Specific energy conductance decomposer	Eichinger *et al.* (2009)
$\dot {h}$	d$^{-1}$	0.01	Instantaneous death rate consumer	-
$\kappa _{E1}$	-	0.7	Return fraction of rejected producer reserve 1	Lorena *et al.* (2010)
$\kappa _{E2}$	-	0.7	Return fraction of rejected producer reserve 2	Lorena *et al.* (2010)
$\rho $	-	0.01	Preference within demand-driven preference SU	
$T_{min}$	$^\circ $ C	2.85	Annual minimum daily temperature	Deltares (2021)
$T_{max}$	$^\circ $ C	19.6	Annual maximum daily temperature	Deltares (2021)
$T_{A}$	K	8000	Arrhenius temperature	
$T_{1}$	K	293	Reference temperature	
$t_{Tmax}$	d	225	Day of year of maximum temperature	Deltares (2021)
$\dot {J}_{L,min}$	MJ m$^{-2}$ d$^{-1}$	0.98	Annual minimum daily irradiance	KNMI (2021)
$\dot {J}_{L,max}$	MJ m$^{-2}$ d$^{-1}$	20.88	Annual maximum daily irradiance	KNMI (2021)
$t_{\dot {J}_{L,max}}$	d	170	Day of year of maximum irradiance	KNMI (2021)
$K_S$	L $\mu $mole$^{-1}$ C	0.1	Self-shading parameter	

**Table 6:** Various conversion efficiencies $y$ and nitrogen content per carbon $n_N$ within the canonical community model.

Name	Unit	Value	Comment	Source
$y_{EC,VP}$	mole C/mole C	0.5	Producer structure to consumer reserves	Muller *et al.* (2001)
$y_{EC,E_1P}$	mole C/mole C	0.8	Producer reserves 1 to consumer reserves	Kooijman & Nisbet (2000)
$y_{EC,E_2P}$	mole C/mole C	0.8	Producer reserves 2 to consumer reserves	Kooijman & Nisbet (2000)
$y_{PP,VP}$	mole C/mole C	0.5	Producer structure to faeces	Assumed $1-y_{EC,VP}$
$y_{EC,VD}$	mole C/mole C	0.5	Decomposer structure to consumer reserves	Muller *et al.* (2001)
$y_{EC,ED}$	mole C/mole C	0.8	Decomposer reserves to consumer reserves	Kooijman & Nisbet (2000)
$y_{PD,VD}$	mole C/mole C	0.5	Decomposer structure to detritus pool 2	Assumed $1-y_{EC,VD}$
$y_{ED,PP}$	mole C/mole C	0.5	Detritus 1 to decomposer reserves	Eichinger *et al.* (2009)
$y_{ED,PD}$	mole C/mole C	0.5	Detritus 2 to decomposer reserves	Eichinger *et al.* (2009)
$y_{ED,PV}$	mole C/mole C	0.5	Detritus 3 to decomposer reserves	Eichinger *et al.* (2009)
$y_{ED,PE}$	mole C/mole C	0.5	Detritus 4 to decomposer reserves	Eichinger *et al.* (2009)
$y_{EC,VC}$	mole C/mole C	1.237	Consumer structure to consumer reserves	Kooijman & Gergs (2019)
$y_{E_1P,VP}$	mole C/mole C	1.25	Producer structure to producer reserves 1	Lorena *et al.* (2010)
$y_{E_2P,VP}$	mole C/mole C	1.5	Producer structure to producer reserves 2	Lorena *et al.* (2010)
$y_{ED,VD}$	mole C/mole C	1.492	Decomposer structure to decomposer reserves	Eichinger *et al.* (2009)
$n_{N,PP}$	mole N/mole C	0.15	Detritus 1	Equal to $n_{N,VP}$
$n_{N,PD}$	mole N/mole C	0.15	Detritus 2	Equal to $n_{N,VD}$
$n_{N,PV}$	mole N/mole C	0.15	Detritus 3	Equal to $n_{N,VC}$
$n_{N,PE}$	mole N/mole C	0.15	Detritus 4	Equal to $n_{N,EC}$
$n_{N,VC}$	mole N/mole C	0.15	Consumer structure	Martiny *et al.* (2014)
$n_{N,EC}$	mole N/mole C	0.15	Consumer reserves	Martiny *et al.* (2014)
$n_{N,VP}$	mole N/mole C	0.15	Producer structure	Martiny *et al.* (2014)
$n_{N,E_1P}$	mole N/mole C	0	Producer reserves 1	-
$n_{N,E_2P}$	mole N/mole C	0.8	Producer reserves 2	
$n_{N,VD}$	mole N/mole C	0.15	Decomposer structure	Martiny *et al.* (2014)
$n_{N,ED}$	mole N/mole C	0.15	Decomposer reserves	Martiny *et al.* (2014)

For completeness, the equations can be written out explicitly. The dissipation flux from the reserves, which is equivalent to minus the rate of maintenance costs paid from reserves, is thus given by

(17) \begin{align*}& \dot{J}_{EC,DC}=-\dot{J}_{EC}^{MC}=\frac{\dot{k}\dot{J}_{EC,CC}}{\dot{k}-\dot{J}_{EC,CC}} \end{align*}

with $\dot {k}$ as above and

\begin{eqnarray*} A&=&\rho\left(-\dot{J}_{EC,CC}-y_{EC,VC}\dot{J}_{VC,CC}-\dot{J}_{EC,MC}\right)\\ B&=& -\dot{J}_{EC,CC}(-\dot{J}_{EC,CC}-y_{EC,VC}\dot{J}_{VC,CC})-(-\dot{J}_{EC,CC}\rho\\&&
-\dot{J}_{EC,CC}-y_{EC,VC}\dot{J}_{VC,CC})\dot{J}_{EC,MC}\\ C&=&\dot{J}_{EC,CC}(-\dot{J}_{EC,CC}-y_{EC,VC}\dot{J}_{VC,CC})\dot{J}_{EC,MC}, \end{eqnarray*}

where $\rho $ is a preference parameter.

Note that the overall maintenance costs are the sum of the costs paid from reserves and those paid from structure, which then directly gives the maintenance costs paid from structure, in terms of C-moles reserve,

\begin{align*} \dot{J}_{EC,MC}=y_{EC,VC}\dot{J}_{VC}^{MC}+\dot{J}_{EC}^{MC}. \end{align*}

Recall that all these maintenance costs, $\dot {J}_{VC}^{MC}$, $\dot {J}_{EC}^{MC}$ and $\dot {J}_{EC,MC}$, are presented as positive fluxes. Consequently, the dissipation flux from structure, which is equivalent to the part paid from structure, equals, in C-moles of structure

(18) \begin{align*}& \dot{J}_{VC,DC}=-\dot{J}_{VC}^{MC}=-(\dot{J}_{EC,MC}-\dot{J}_{EC}^{MC})/y_{EC,VC}. \end{align*}

Alternatively, the maintenance costs paid from structure could have been calculated (using the notation of the preference unit as used in Preference SU section) as

\begin{align*} y_{EC,VC}\dot{J}_{VC}^{MC}=\frac{y_2\rho\dot{k}^2\dot{J}_2}{(\dot{k}+\dot{J}_1)(\rho\dot{k}+\dot{J}_1+\dot{J}_2)} \end{align*}

but this gives, of course, exactly the same result.

The rejected flux from the structure (which equals -$\dot {J}_{VC,CC}-\dot {J}_{VC}^{MC}$) is immediately channeled back to the structure. The rejected flux from the reserves is allocated to growth, and the flux from reserves to growth therefore equals

(19) \begin{align*}& \dot{J}_{EC,GC}=-(-\dot{J}_{EC,CC}-\dot{J}_{EC}^{MC}), \end{align*}

which results in an increase of structure equal to

(20) \begin{align*}& \dot{J}_{VC,GC}=-\dot{J}_{EC,GC}/y_{EC,VC}. \end{align*}

The overall mass balance of the structure is thus given by a positive incoming flux $\dot {J}_{VC,GC}$ and a negative outgoing flux $\dot {J}_{VC}^{MC}$. The overall result can be simplified to

\begin{align*} \dot{J}_{VC,GC}-\dot{J}_{VC}^{MC}&=\frac{-\dot{J}_{EC,CC}-\dot{J}_{EC}^{MC}-y_{EC,VC}\dot{J}_{VC}^{MC}}{y_{EC,VC}}\\&=\frac{-\dot{J}_{EC,CC}-\dot{J}_{EC,MC}}{y_{EC,VC}}. \end{align*}

So in fact knowing $\dot {J}_{EC,CC}$ and $\dot {J}_{EC,MC}$ is sufficient for describing the mass balance of the structure. One might therefore argue that the idea of a preference SU is not really needed. One simply can accept that a negative growth flux from structure to reserves is possible. However, for producers, which are discussed below and which have multiple reserves, such simplification is not possible and preference SUs are needed to describe maintenance and growth.

As shown above, consumers will not die from starvation, for example when maintenance costs cannot be met as a result of too low reserve density, but will then also use structure to pay for maintenance and consequently shrink. In addition, we assumed a natural background mortality, where the death rate depends upon the reserve density. The death of consumers results in a flux from consumer structure to the dead consumer structure part of the detritus pool, given by

(21) \begin{align*}& \dot{J}_{PV,HC}=-\dot{J}_{VC,HC}=\dot{h}_aM_{VC}\frac{m_{EC}/y_{EC,VC}}{1+m_{EC}/y_{EC,VC}} \end{align*}

and in a similar flux of consumer reserves to the dead consumer reserve part of the detritus pool

(22) \begin{align*}& \dot{J}_{PE,HC}=-\dot{J}_{EC,HC}=m_{EC}\dot{J}_{PV,HC}. \end{align*}

### 4.2 Producers

The producers have two types of reserves. The first type contains only carbohydrates and the assimilation rate for this type depends upon the light conditions and the carbon-compound concentration. Using an SU with two non-substitutable substrates gives the assimilation rate

(23) \begin{align*}& \dot{J}_{E_1P,A_1P}=M_{VP}j_{E_1P,A_1P,m}f_{P_1}, \end{align*}

where

\begin{align*} f_{P_1}=\left(1+\frac{1}{x_{L_1}}+\frac{1}{x_{C_1}}-\frac{1}{x_{L_1}+x_{C_1}}\right)^{-1}, \end{align*}

where $x_{L_1}=\dot {J}_L/\dot {J}_{K,L_1}$ and $x_{C_1}=M_C/K_{C_1}$. The assimilation rate of the other reserve type depends upon light, the carbon-compound and ammonium and is (using a SU with three non-substitutable substrates) given by

(24) \begin{align*}& \dot{J}_{E_2P,A_2P}=M_{VP}j_{E_2P,A_2P,m}f_{P_2}, \end{align*}

where

\begin{align*} f_{P_2}&=\left(1+\frac{1}{x_{L_2}}+\frac{1}{x_{C_2}}+\frac{1}{x_{N_2}}-\frac{1}{x_{L_2}+x_{C_2}}-\frac{1}{x_{L_2}+x_{N_2}}\right.\\
&\quad\left.\quad -\frac{1}{x_{C_2}+x_{N_2}}+\frac{1}{x_{L_2}+x_{C_2}+x_{N_2}}\right)^{-1}, \end{align*}

where $x_{L_2}=\dot {J}_L/\dot {J}_{K,L_2}$, $x_{C_2}=M_C/K_{C_2}$ and $x_{N_2}=M_N/K_{N_2}$.

From each of the two reserves, a mobilized flux, which equals

\begin{align*} \dot{J}_{E_iP,CP}=-m_{E_iP}(\dot{k}_{E_iP}-\dot{r}_{VP,GP})M_{VP} \end{align*}

is, together with a mobilized flux from the structure, which expressed in C-moles reserve equals

\begin{align*} y_{E_iP,VP}\dot{J}_{V_iP,CP}=-y_{E_iP,VP}j_{V_iP,CP}M_{VP} \end{align*}

directed towards a demand-driven preference SU. So there are two of these SUs. Each of these two units generates a maintenance flux with contributions from both reserves and structure

\begin{align*} \dot{J}_{E_iP,MP}=\dot{J}_{E_iP}^{MP}+y_{E_iP,VP}\dot{J}_{V_iP}^{MP}. \end{align*}

The maintenance that is paid from the reserves $\dot {J}_{E_iP}^{MP}$ is calculated in a similar way as for the consumers (i.e. as explained above). This also holds for the maintenance paid from structure, which is given by $y_{E_iP,VP}\dot {J}_{V_iP}^{MP}$. The rejected fluxes from the mobilized fluxes from structure are directly channeled back to the structure. Hence, these fluxes do not play a role in the mass balance. The two rejected fluxes from the mobilized fluxes from the reserves are now first directed towards a growth SU, which is a SU at which two non-substitutable substrates arrive. These two fluxes equal, in terms of C-moles of structure,

\begin{align*} \dot{J}_{E_iP,GP}/y_{E_iP,VP}=(-\dot{J}_{E_iP,CP}-\dot{J}_{E_iP}^{MP})/y_{E_iP,VP}. \end{align*}

The product of this growth SU represents the overall growth flux and is given by

\begin{align*} \dot{J}_{VP,GP}&=\left(\frac{1}{\dot{J}_{E_1P,GP}/y_{E_1P,VP}}+\frac{1}{\dot{J}_{E_2P,GP}/y_{E_2P,VP}}\right.\\
&\qquad\left.-\frac{1}{\dot{J}_{E_1P,GP}/y_{E_1P,VP}+\dot{J}_{E_2P,GP}/y_{E_2P,VP}}\right)^{-1}. \end{align*}

The overall growth rate equals

\begin{align*} \dot{r}_{VP,GP}=\frac{\dot{J}_{VP,GP}-\dot{J}_{V_1P}^{MP}-\dot{J}_{V_2P}^{MP}}{M_{VP}}. \end{align*}

Note that the three terms in the numerator at the right side of the equation depend upon the growth rate $\dot {r}_{VP,GP}$ as well and no explicit equation for the growth rate $\dot {r}_{VP,GP}$ is available. A root-finding procedure has to be used to calculate the growth rate.

The flux towards growth thus equals

(25) \begin{align*}& \dot{J}_{VP,GP}=\dot{r}_{VP,GP}M_{VP}+\dot{J}_{V_1P}^{MP}+\dot{J}_{V_2P}^{MP}, \end{align*}

and the contribution of each of the two reserves is

(26) \begin{align*}& \dot{J}_{E_iP}^{GP}=-y_{E_iP,VP}\dot{J}_{VP,GP} \end{align*}

Recall that the two types of reserves arriving at the growth SU are non-substitutable substrates, and the substrate that is non-limiting at arrival will be rejected. A fraction $\kappa _{E_i}$ of the rejected flux is channeled back into the reserves, the rest is dissipated. Each rejected flux equals

\begin{align*} \dot{J}_{E_iP,GP}-\dot{J}_{E_iP}^{GP}. \end{align*}

The total dissipation flux from the reserves, which also includes the maintenance paid from the reserves, is therefore equal to

(27) \begin{align*}& \dot{J}_{E_iP,DP}= (1-\kappa_{Ei})(\dot{J}_{E_iP,GP}-\dot{J}_{E_iP}^{GP})-\dot{J}_{E_iP}^{MP}. \end{align*}

The dissipation flux from the structure equals the maintenance costs paid from the structure (in units of structure)

(28) \begin{align*}& \dot{J}_{V_iP,D_iP}=-\dot{J}_{V_iP}^{M_iP}. \end{align*}

**Fig. 6:**
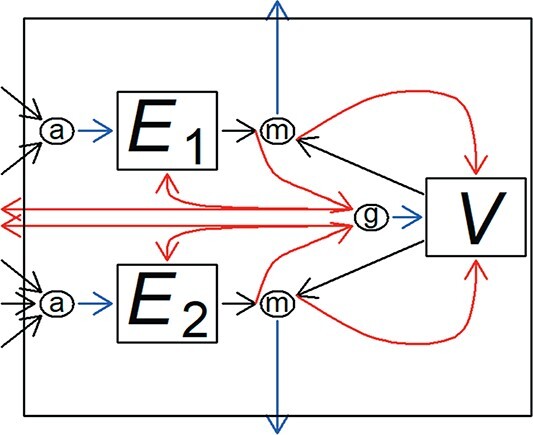
Mass fluxes through the producer. Squares represent the two reserve compartments ($E_1$ and $E_2$) and the structural volume ($V$). SUs are presented by circles. Nutrients and light arrive at two assimilation SUs, whose products enter the reserves (product flows are shown by blue arrows). For each of the reserves, the mobilized flux is, together with a flux from the structure, directed towards a maintenance SU. The products of these SUs are dissipated. The rejected fluxes are shown by red arrows. Those originating from the structural volume are sent back, and those originating from the reserves go to a growth SU, whose product is channeled to the structural volume. Fluxes rejected by the growth unit are partly sent back to the reserves, the rest dissipates. Nutrient fluxes rejected by the assimilation SUs are not depicted.

Summarizing, nutrients and light arrive at two SUs that handle non-substitutable substrates. The products are channeled to two reserve types. For each of the reserves, the mobilized flux is, together with a flux from the structure, directed towards a demand driven preference SU, whose product is used for maintenance. The rejected fluxes originating from the two reserves go to a growth SU, which handles two non-substitutable substrates. All together, five SUs are thus needed to describe the mass fluxes through the producer (Fig. 6).

### 4.3 Decomposers

The decomposers live on detritus and the removal rate for each of the four types of detritus follows a multiple prey type II functional response (SU with four substitutable substrates)

(29) \begin{align*}& \dot{J}_{*,A_iD}=-j_{*,A_iD,m}\frac{x_*}{1+x_{PP}+x_{PD}+x_{PV}+x_{PE}}M_{VD} \end{align*}

for $(i,*)\in \{(1,PP),(2,PD),(3,PV),(4,PE)\}$. The consumed material is assimilated and enters the reserves of the decomposers with a rate equal to

(30) \begin{align*}& \dot{J}_{ED,A_iD}=-y_{ED,*}\dot{J}_{*,A_iD} \end{align*}

again for for $(i,*)\in \{(1,PP),(2,PD),(3,PV),(4,PE)\}$. Just as for the consumers, the reserves and structure of the decomposers are mobilized and directed towards a demand-driven preference SU. The rejected flux from the structure is channeled back to the structure, and the rejected flux from the reserves is allocated to growth. Hence,

(31) \begin{align*}& \dot{J}_{ED,GD}=-\big(-\dot{J}_{ED,CD}-\dot{J}_{ED}^{MD}\big) \end{align*}

(32) \begin{align*}& \dot{J}_{VD,GD}=-\dot{J}_{ED,GD}/y_{ED,VD} \end{align*}

(33) \begin{align*}& \dot{J}_{ED,DD}=-\dot{J}_{ED}^{MD} \end{align*}

(34) \begin{align*}& \dot{J}_{VD,DD}=-\dot{J}_{VD}^{MD}=-\big(j_{ED,MD}M_{VD}-\dot{J}_{ED}^{MD}\big)/y_{ED,VD}, \end{align*}

where $\dot {J}_{ED}^{MD}$ is calculated in the same way as explained for the consumers.

### 4.4 Overall model

The system of differential equations follows directly from the flux matrix $\dot {J}$ and mass conservation considerations

\begin{eqnarray*} \frac{\textrm{d}}{\textrm{d}t}M_{PP}&=&\dot{J}_{PP}=\dot{J}_{PP,A_1C}+\dot{J}_{PP,A_1D}\\ \frac{\textrm{d}}{\textrm{d}t}M_{PD}&=&\dot{J}_{PD}=\dot{J}_{PD,A_2C}+\dot{J}_{PD,A_2D}\\ \frac{\textrm{d}}{\textrm{d}t}M_{PV}&=&\dot{J}_{PV}=\dot{J}_{PV,HC}+\dot{J}_{PV,A_3D}\\ \frac{\textrm{d}}{\textrm{d}t}M_{PE}&=&\dot{J}_{PE}=\dot{J}_{PE,HC}+\dot{J}_{PE,A_4D}\\ \frac{\textrm{d}}{\textrm{d}t}M_{VC}&=&\dot{J}_{VC}=\dot{J}_{VC,GC}+\dot{J}_{VC,DC}+\dot{J}_{VC,HC}\\ \frac{\textrm{d}}{\textrm{d}t}M_{EC}&=&\dot{J}_{EC}=\!\sum_{i=1}^2{\dot{J}_{EC,A_iC}}\!+ \dot{J}_{EC,GC}\!+\dot{J}_{EC,DC}\!+\dot{J}_{EC,HC}\\ \frac{\textrm{d}}{\textrm{d}t}M_{VP}&=&\dot{J}_{VP}=\dot{J}_{VP,A_1C}+\dot{J}_{VP,GP}+\dot{J}_{VP,DP}\end{eqnarray*}

\begin{eqnarray*} \frac{\textrm{d}}{\textrm{d}t}M_{E_1P}&=&\dot{J}_{E_1P}=\dot{J}_{E_1P,A_1C}\!+\dot{J}_{E_1P,A_1P}\!+ \dot{J}_{E_1P}^{GP}\!+\dot{J}_{E_1P,DP}\\ \frac{\textrm{d}}{\textrm{d}t}M_{E_2P}&=&\dot{J}_{E_2P}=\dot{J}_{E_2P,A_1C}\!+\dot{J}_{E_2P,A_2P}\!+ \dot{J}_{E_2P}^{GP}\!+\dot{J}_{E_2P,DP}\\ \frac{\textrm{d}}{\textrm{d}t}M_{VD}&=&\dot{J}_{VD}=\dot{J}_{VD,A_2C}+\dot{J}_{VD,GD}+\dot{J}_{VD,DD}\\ \frac{\textrm{d}}{\textrm{d}t}M_{ED}&=&\dot{J}_{ED}\!=\!\dot{J}_{ED,A_2C}\!+\!\sum_{i=1}^4{\dot{J}_{ED,A_iD}}\!+\! \dot{J}_{ED,GD}\!+\dot{\!J}_{ED,DD}\\ \frac{\textrm{d}}{\textrm{d}t}M_{C}&=&\dot{J}_{C}=-\sum_*{\dot{J}_{*}}\\ \frac{\textrm{d}}{\textrm{d}t}M_{N}&=&\dot{J}_{N}=-\sum_*{n_{N,*}\dot{J}_{*}} \end{eqnarray*}

for $*\in \{PP,PD,PV,PE,VC,EC,VP,E_1P,E_2P,VD,ED\}$.

### 4.5 Temperature and light

DEB theory assumes that all rates respond in the same way to temperature (Kooijman, 2000). The Van’t Hoff-Arrhenius equation is used to correct rates for temperature

\begin{align*} \dot{k}(T)=\dot{k}_1\exp\left(\frac{T_A}{T_1}-\frac{T_A}{T}\right), \end{align*}

where $\dot {k}(T)$ is the reaction rate that depends upon the absolute temperature $T$ (in Kelvin), $\dot {k}_1$ is the reaction rate at a reference temperature $T_1$ and $T_A$ is the Arrhenius temperature, assumed to be constant within the same organism. Temperature corrections can be added to the main equations by multiplying all rates by the Van’t Hoff-Arrhenius correction factor.

In the canonical ecosystem model, seasonal fluctuation in temperature is modelled with a cosine function that varies between the minimum temperature $T_{min}$ ($^{\circ }$C) and maximum temperature $T_{max}$ ($^{\circ }$C), which is reached at day of the year $t_{Tmax}$:

(35) \begin{align*} T(t) &= 273.15 + T_{min} + 0.5 \left( T_{max} - T_{min} \right)\notag \\&\quad\cdot \left( 1+ \textrm{cos} \left( (t - t_{Tmax}) \frac{2\pi}{365} \right) \right). \end{align*}

The constant 273.15 converts temperature in degree Celsius to Kelvin, as required by the Van ’t Hoff-Arrhenius function.

Yearly fluctuation in light irradiation ($\dot {J}_{L_{in}}$ in MJ m$^{-2}$ day$^{-1}$) is modelled similar to yearly fluctuation in temperature, but using different parameters:

(36) \begin{align*} \dot{J}_{L_{in}}(t) &= \dot{J}_{L,min} + 0.5 \left( \dot{J}_{L,max} - \dot{J}_{L,min} \right)\notag\\&\quad \cdot \left( 1+ \textrm{cos} \left( \left(t - t_{ \dot{J}_{L,max}} \right) \frac{2\pi}{365} \right) \right). \end{align*}

We assumed that light availability decreases through self shading as a function of producer structural mass. Light availability including self-shading is given by

(37) \begin{align*}& \dot{J}_L(t) = \dot{J}_{L_{in}}(t) \exp\left(K_S M_{VP}\right). \end{align*}

Parameter $K_S$ modifies the strength of self-shading by the producer.

### 4.6 Parameterization

All model parameters and their sources are listed in Tables 5 and 6. Where possible we refined the original parameterization of Kooijman & Nisbet (2000). Producer parameters were mainly taken from Lorena *et al.* (2010), who proposed a DEB model for a general microalgae. Producer specific energy conductance parameters $\dot {k}_{E_1P}$ and $\dot {k}_{E_2P}$ were refined by Grossowicz *et al.* (2017). Parameters for the decomposer were taken from Eichinger *et al.* (2009), who presented a DEB model for marine bacterial biomass. Parameters related to the decomposer’s functional response were taken from Lorkowski *et al.* (2012).

Consumers are for reasons of convenience treated as V1-morphs in the canonical ecosystem model, although most zooplankton and zoobenthos species are actually better characterized as isomorphs. V1-morph parameters can be derived from isomorph parameters by assuming that all individuals have the same reference structural volume $V_d$. The maximum volume-specific assimilation rate can then be obtained from the maximum area-specific assimilation rate as follows: [*ṗ*_*Am*_] = {*ṗ*_*Am*_}*V*_*d*_^−1/3^ . Similarly, the specific energy conductance parameter $\dot {k}_E$ can be obtained as *k̇*_*E*_ = *v̇V*_*d*_^−1/3^ , where $\dot {v}$ (cm d$^{-1}$) is the primary DEB parameter that describes the energy conductance of an isomorph. Subsequently, the energy-length parameters for V1-morphs can be converted to mass–mass parameters by dividing each volume-specific parameter by the energy density of structure $\mu _E[M_V]$ in J cm$^{-3}$.

We derived V1 parameters for consumers from the isomorphic parameters for *Daphnia magna* available in the Add-my-Pet library (Kooijman & Gergs, 2019). We choose the length at puberty for *D. magna* as reference structural length ($L_d = V_d^{1/3} = 0.07$ cm) and a value of $\mu _E[M_V]$ equal to 3556 J cm$^{-3}$ (Table 4). The obtained mass-specific maximum assimilation was divided by the estimated digestion efficiency of food to reserve ($\kappa _X = 0.9$) to obtain a mass-specific maximum consumption rate ($j_{EAm}$). We assumed that in the canonical ecosystem model, maximum consumption of producer structure equals $j_{EAm}$, while maximum consumption of decomposer structure by consumers equals $0.5 j_{EAm}$. Consumer mortality was set at $h_C = 0.01$.

Parameters that describe the nitrogen content per carbon were taken as much as possible equal to the observed global median C:N ratio of 6.6 (‘Redfield ratio’) as described for phytoplankton (Martiny *et al.*, 2014). Nitrogen content of the carbon and the nitrogen reserves of the producer were set to 0 and 0.8, respectively.

Temperature parameters were obtained from data on water temperatures in the North Sea from 2004 onwards at the measurement station of Schiermonnikoog Noord, The Netherlands (Deltares, 2021). Irradiance parameters were derived from measured irradiance from 2004 onwards at De Kooy, a measurement station of the Royal Netherlands Meteorological Institute (KNMI, 2021).

### 4.7 Implementation

The model was implemented in R software (R Core Team, 2021) using the R-package deSolve (Soetaert *et al.*, 2010). The code to run the analysis is available via the Open Science Framework (https://osf.io/nfqgh/).

### 4.8 Dynamics

**Fig. 7:**
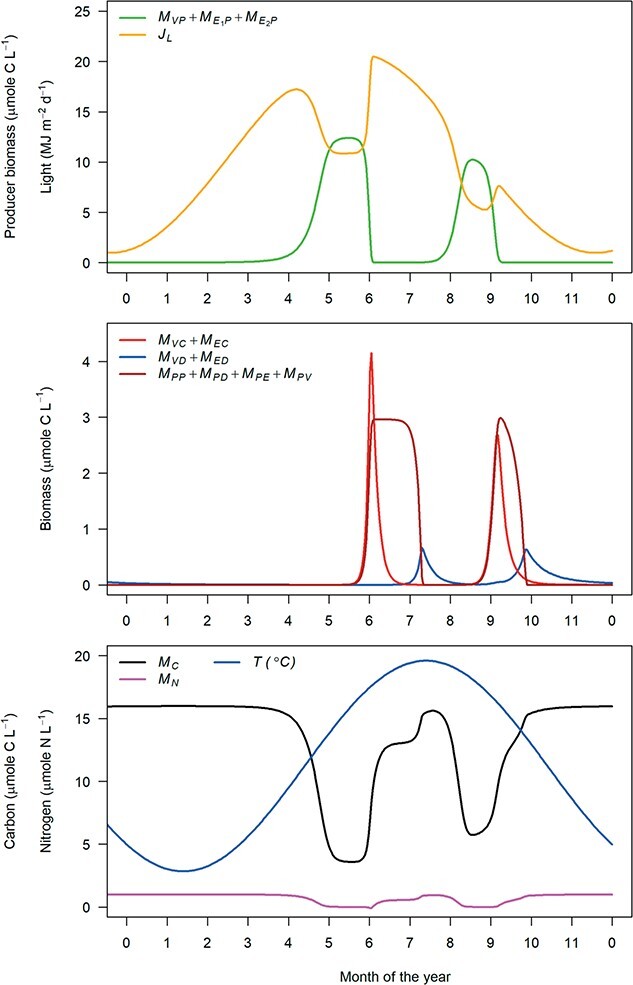
Dynamics of the canonical ecosystem model in the yearly repeating stable attractor. All parameters as in Tables 5 and 6. Plotted in the last year of a simulation over 5 years (1825 days) with initial values $M_{PP}(0) = $ 2.66E-2, $M_{PD}(0) = $ 7.986E-3, $M_{PV}(0) = $ 2.66E-04, $M_{PE}(0) = $ 6.66E-06, $M_{VC}(0) = $ 1.02E-01, $M_{EC}(0) = $ 6.66E-03, $M_{VP}(0) = $ 3.99E-03, $M_{E_1P}(0) = $ 3.99E-03, $M_{E_2P}(0) = $ 3.99E-03, $M_{VD}(0) = $ 2.66E-03, $M_{ED}(0) = $ 1.33E-03, $M_{C}(0) = $ 1.58E01 and $M_{N}(0) = $ 9.74E-01.

The dynamics of the canonical ecosystem model converge towards a stable attractor with two distinct producer (phytoplankton) peaks per year; one in April/May and a slightly lower and shorter peak in August (Fig. 7). Both producer peaks are followed by an increase in consumer biomass. Because the growth of producer biomass has already ceased before consumer biomass increases, the producer growth is limited by nutrient and light availability, which decrease due to assimilation and self-shading. However, consumers are responsible for the subsequent decrease in producer biomass, because producer background mortality is not accounted for. Because all detritus pools are associated with the consumer either through feeding (faeces: $M_{PP}$ and $M_{PD}$) or dead consumer biomass ($M_{PV}$ and $M_{PE}$), detritus biomass increases alongside the growth of consumers. Upon suppression of producer biomass, consumers die from starvation and background mortality and the detritus is cleared by decomposers.

## 5 Discussion

We present a minimum ecosystem model based on DEB theory, with an example mimicking a marine ecosystem. The model is described in a mass–mass framework, as opposed to the more conventional energy-length DEB framework (Kooijman, 2010), and explicitly considers nitrogen and carbon stoichiometry. All three functional groups, that is producers, consumers and decomposers, are treated as V1-morphs and either contain one (consumers and decomposer) or two (producers) reserve compartments. Different types of SUs regulate substrate uptake, maintenance and growth. For the marine ecosystem application the producer represents the community of phytoplankton species, the consumer represents the zooplankton species and the decomposer represents the bacteria. Dynamics are driven both by environmental variables and species interactions. The set of parameters and simulations was presented for a part of the North Sea ecosystem, along the Dutch coastline.

Under a seasonally fluctuating temperature and light regime, model dynamics show a spring and autumn peak of primary production (Daewel *et al.*, 2014, Maar *et al.*, 2018). A bimodal seasonal pattern of phytoplankton has been observed in coastal waters such as the North Sea (Cadéee, 1986, Colebrook, 1982, 1984, Desmit *et al.*, 2020, Pitois *et al.*, 2012, Reid *et al.*, 1990) and is reproduced by several high-resolution ecosystem models of lower-trophic-level processes (Los *et al.*, 2008). Simulated consumer biomass also shows two peaks directly following the peaks in primary production. Despite large spatio-temporal variation, seasonal dynamics of zooplankton abundance in the North Sea typically show a unimodal pattern with high zooplankton biomass from spring until autumn (Colebrook, 1984, Fransz *et al.*, 1991, Pitois *et al.*, 2012, Van de Wolfshaar *et al.*, 2021), although localized bimodal patterns in zooplankton have been observed in the North Sea (Colebrook, 1984). A potential reason for the difference between generally observed and modelled zooplankton seasonal dynamics is the strong coupling between producers and consumers in our model. Further explorations should reveal whether a different parameter setting can dampen the strong producer–consumer interaction, or that additional mechanisms, such as for example consumer interference or extra producer or consumer functional groups are required for a more sustained peak of consumer biomass. Simulated decomposer biomass shows two delayed peaks, but data to confirm whether such pattern occurs in the field are lacking.

Because the presented ecosystem model is based on a formal theory about metabolic organization (DEB), modelled processes and their parameters directly relate to processes at the individual level. This approach differs fundamentally from most marine ecosystem models of lower trophic levels (for an overview, see Maar *et al.*, 2018), which often rely on ad hoc descriptions of population or ecosystem processes (Marques *et al.*, 2014). In contrast, DEB provides a quantitative approach built on a collection of assumptions and stylized facts concerning individual-level energy acquisition, allocation and use and is well-tested across a wide range of taxa (Lorena *et al.*, 2010, Sousa *et al.*, 2008, 2010, van der Meer *et al.*, 2014).

The use of DEB for ecosystem modelling also allows extending our minimum ecosystem model with additional food web components, as organismal DEB models and their parameters are readily available (Marques *et al.*, 2018, van der Meer, 2006a, van der Meer *et al.*, 2014). We provide an example on how to relate ‘energy-length’ DEB parameters for isomorphs to the ‘mass–mass’ parameters for V1-morphs as used in the present ecosystem model. If the V1-morph approach is deemed inappropriate, which is probably the case for higher trophic levels, a cohort-based approach could be adopted (van der Meer, 2016). However, this would considerably complicate model implementation and analysis. Future studies should shed more light on how system dynamics is affected by the assumption of V1-morphy for organisms that show a much larger size range during ontogeny than isomorphs that simply divide into two daughter cells. In addition, a spatial component can easily be incorporated through a coupled hydrodynamic model that simulates the transport of nutrients and pelagic ecosystem components across distinct spatial compartments. As such, an extended version of the model presented here would provide a powerful tool for studying a wide variety of questions related to marine conservation and management, such as ecosystem effects of human impacts (e.g. eutrophication, fishing), potential for mariculture and marine spatial planning. The use of the mass–mass framework allows to use stoichiometry of the different food web components to keep track of and maintain mass balance of nutrients such as nitrogen and phosphorous. In the example presented here, phosphorous is not included, but it can be added to address questions on for example phosphorous and nitrogen limitation in response to (terrestrial) management actions.

In its current form, the present model sacrifices realism to precision and generality (Levins, 1966). This contrasts most ecosystem models of lower trophic levels, which have high spatial and temporal resolution and consider multiple functional phytoplankton and zooplankton groups in order to maximize correspondence with data and by doing so try to achieve high predictive ability and thus realism. Due to their long computation times, these models rarely address parameter uncertainties nor study how the obtained results depend on model assumptions (Rose *et al.*, 2010). This can more readily be achieved by simpler and more general models, for which a more complete sensitivity analysis is within reach. In addition, models like the present one can be used to explore how various ecosystem processes, such as the mode of tropho-dynamic control or the processes that limit phytoplankton and zooplankton growth, depend on various model parameters and assumptions.

**Authorship statement:** J.v.d.M. and V.H. wrote the first draft. All others contributed to the writing. J.v.d.M. and V.H. wrote the model code, with contributions from P.v.O. V.H. collected all parameter values.

**Data accessibility statement:** No original data have been used.
